# Low PI-RADS assessment category excludes extraprostatic extension (≥pT3a) of prostate cancer: a histology-validated study including 301 operated patients

**DOI:** 10.1007/s00330-019-06092-0

**Published:** 2019-03-18

**Authors:** Sarah Alessi, Paola Pricolo, Paul Summers, Marco Femia, Elena Tagliabue, Giuseppe Renne, Roberto Bianchi, Gennaro Musi, Ottavio De Cobelli, Barbara Alicja Jereczek-Fossa, Massimo Bellomi, Giuseppe Petralia

**Affiliations:** 1 Division of Radiology, IEO European Institute of Oncology IRCCS, Via Ripamonti 435, 20141 Milan, Italy; 2 Postgraduation School in Radiodiagnostics, Università degli Studi di Milano, Via Festa del Perdono 7, Milan, 20122 Italy; 3 Multimedica IRCCS, Milan, Italy; 4 Department of Pathology, IEO European Institute of Oncology IRCCS, via Ripamonti 435, Milan, 20141 Italy; 5 Department of Urology, IEO European Institute of Oncology IRCCS, Via Ripamonti 435, 20141 Milan, Italy; 6 Division of Radiation Oncology, IEO European Institute of Oncology IRCCS, Via Ripamonti 435, 20141 Milan, Italy; 7 Department of Oncology and Hemato-oncology, University of Milan, Via Festa del Perdono 7, Milan, 20122 Italy

**Keywords:** Prostate cancer, Magnetic resonance imaging, Nomogram

## Abstract

**Objectives:**

To evaluate whether low PI-RADS v2 assessment categories are effective at excluding extraprostatic extension (EPE) of prostate cancer (≥pT3a PCa).

**Methods:**

The local institutional ethics committee approved this retrospective analysis of 301 consecutive PCa patients. Patients were classified as low- or intermediate/high-risk based on clinical parameters and underwent pre-surgical multiparametric magnetic resonance imaging. A PI-RADS v2 assessment category and ESUR EPE score were assigned for each lesion by two readers working in consensus. Histopathologic analysis of the whole-mount radical prostatectomy specimen was the reference standard. Univariate and multivariate analyses were performed to evaluate the association of PI-RADS v2 assessment category with final histology ≥pT3a PCa.

**Results:**

For a PI-RADS v2 assessment category threshold of 3, the overall performance for ruling out (sensitivity, negative predictive value, negative likelihood ratio) ≥pT3a PCa was 99%/98%/0.04 and was similar in both the low-risk (96%/97%/0.12; *N* = 137) and the intermediate/high-risk groups (100%/100%/0.0; *N* = 164). In univariate analysis, all clinical and tumor characteristics except age were significantly associated with ≥pT3a PCa. In multivariate analysis, PI-RADS v2 assessment categories ≤ 3 had a protective effect relative to categories 4 and 5. The inclusion of ESUR EPE score improved the AUC of ≥pT3a PCa prediction (from 0.73 to 0.86, *p* = 0.04 in the overall cohort). The impact of PI-RADS v2 assessment category is reflected in a nomogram derived on the basis of our cohort.

**Conclusions:**

In our cohort, low PI-RADS v2 assessment categories of 3 or less confidently ruled out the presence of ≥pT3a PCa irrespective of clinical risk group.

**Key Points:**

*• Our analysis of 301 mp-MRI and RARP specimens showed that the addition of PI-RADS v2 assessment categories to clinical parameters improves the exclusion of ≥pT3a (extraprostatic) prostate cancer.*

*• PI-RADS v2 assessment categories of 1 to 3 are useful for excluding ≥pT3a prostate cancer with a NPV of 98%; such patients can be considered as candidates for less invasive approaches.*

*• The ability to exclude ≥pT3a prostate cancer may improve confidence in choosing nerve-sparing surgery or in avoiding pelvic nodal dissections, and similarly for patients undergoing radiotherapy, in adopting short-course adjuvant hormonal therapy or foregoing prophylactic nodal irradiation.*

**Electronic supplementary material:**

The online version of this article (10.1007/s00330-019-06092-0) contains supplementary material, which is available to authorized users.

## Introduction

The presence of extraprostatic extension (EPE) of disease in prostate cancer (PCa) patients, corresponding to a pathological stage of ≥pT3a at final histology,^1^ decreases overall and cancer-specific survival following radical prostatectomy (RP) [1]. This has led to interest in predicting the presence or absence of ≥pT3a PCa [2], as a non-invasive technique capable of providing information regarding ≥pT3a PCa at the time of diagnosis could influence decisions regarding treatment. In particular, amongst men with low-risk disease, the absence of ≥pT3a PCa can confirm the suitability of local control (nerve-sparing surgery or radiotherapy) of PCa without a need for adjunct treatment.

Multiparametric magnetic resonance imaging (mp-MRI) is an established imaging technique for PCa detection [3, 4] and has an established role for preoperative staging of PCa [5]. Mp-MRI is also of considerable value in the management of low-risk PCa in men under active surveillance (AS), because it is effective in distinguishing significant from insignificant cancer [6]. A standardized imaging technique and reporting standard for mp-MRI has been created for PCa detection [3] and has evolved into the Prostate Imaging Reporting and Data System version 2 (PI-RADS v2) launched in 2014 [4, 7]. While not specifically designed for the staging of PCa, initial reports by Park et al [8, 9] suggest PI-RADS v2 has potential for predicting ≥pT3a PCa in the preoperative setting. An extraprostatic extension score (ESUR EPE score) has also been defined under ESUR guidelines for MRI [3] but is not explicitly incorporated into the PI-RADS v2 criteria.

The purpose of our study was to evaluate whether low PI-RADS v2 assessment categories are effective at excluding EPE (≥pT3a) of PCa.

## Materials and methods

This retrospective analysis was approved by our institution’s ethics committee, who waived the requirement for a specific informed consent for the study as all patients had given separate written, informed consents for the performance of MRI, for the surgical procedures, and for the use of their clinical data for research purposes.

### Patients

Inclusion criteria were as follows: (a) biopsy-confirmed PCa, (b) mp-MRI, and (c) robotic-assisted radical prostatectomy (RARP) performed at our institution based on clinical-radiological indications or elective choice.

The exclusion criteria were as follows: contraindications for MRI, and previous treatments or the assumption of 5a-reductase inhibitors that could affect the performance of mp-MRI or of final histology.

During the period of this retrospective study (July 2012 and August 2013), 638 patients underwent mp-MRI at our institution, of whom 308 underwent RARP surgery on the basis of clinical findings and personal health management decisions. The time interval between biopsy and mp-MRI ranged from 20 to 50 days, while the time between mp-MRI and RARP ranged from 1 to 3 months.

### mp-MRI technique

PI-RADS v2–compliant prostate mp-MRI was performed on a 1.5-T MR scanner (Avanto, Siemens Medical Solutions). Anterior body (18 channel) and spinal (32 channel) phased-array coils were used without endorectal coil, providing consistently good image quality. The mp-MRI protocol (Supplementary Table 1) involved sagittal, coronal, and axial T2-weighted images; axial diffusion-weighted and pre-contrast T1-weighted images; and a dynamic series of axial T1-weighted images obtained before, during, and after injection of contrast agent (Magnevist, Bayer HealthCare).

### mp-MRI analysis

Two radiologists with respectively 3 and 2 years of experience in mp-MRI of the prostate retrospectively read the images for each patient separately, assigning a PI-RADS v2 assessment category [4] for each lesion, and an ESUR EPE score for any lesion in contact with the prostate capsule [3]. The radiologists were blinded to the original radiological reports and pathological outcomes, but were aware that all patients had PCa, and met for discussion of discordant readings, such that the final PI-RADS v2 categories and ESUR EPE scores were assigned by consensus.

### Surgery

All surgical procedures were performed by surgeons with more than 500 cases’ experience in RARP following an approach based on the technique described by Patel et al [10]. Intraoperative frozen section analysis was performed where the index lesion was considered to have contact with the prostatic capsule, and if the surgical margin was positive, a secondary resection was performed [11].

### Pathology

The prostate total embedding of the whole-mount prostatectomy and any material from secondary resection were classified according to the Gleason scoring system 2005 [12]. Pathologic stage was assigned using the 2009 TNM classification [13], and extraprostatic extension assessed according to “Consensus Prostate Working Group” criteria [14].

### Statistical analysis

Based on pre-imaging clinical characteristics, the patients were divided into three risk groups according to EAU classification [15], but due to the small number of patients in the high-risk group, the intermediate- and high-risk groups were considered together as an “intermediate/high-risk group.”

The radiological variables were considered at the patient level, using the index lesion for each patient; when there were several lesions in the gland, this corresponded to the lesion with the highest PI-RADS score. If there were two lesions with the same PI-RADS score, the lesion with the largest diameter was considered the index lesion. Univariate analyses were performed to evaluate the associations of clinical and radiological variables with pathological stage ≥pT3a. For categorical variable, chi-square or Fisher’s exact tests were used, as appropriate. For the continuous variable “age,” the nonparametric two-sample Wilcoxon test was used, since the Kolmogorov-Smirnov test suggested a non-normal distribution for this variable.

Sensitivity (SE), specificity (SP), positive predictive values (PPV), negative predictive values (NPV), positive likelihood ratio (LR+), and negative likelihood ratio (LR−) for predicting pathological stage ≥pT3a were calculated for the following: clinical risk group, ESUR EPE score, and PI-RADS v2 assessment categories. The diagnostic performance was also evaluated for ESUR EPE score and PI-RADS v2 assessment categories stratified by clinical risk group. For these analyses, ESUR EPE score and PI-RADS v2 assessment categories were analyzed in two classes of cancer likelihood (≤ 3 vs. 4–5), while for the univariate models, they were analyzed in three classes (1–2 vs. 3 vs. 4–5).

Four unconditional logistic regression models for the association with ≥pT3a PCa were evaluated: model 1 included only the clinical risk groups, model 2 added ESUR EPE score to model 1, model 3 added the PI-RADS v2 assessment category to model 1, and model 4 included clinical risk groups, ESUR EPE score, and PI-RADS v2 assessment category. Corresponding odds ratios (ORs) and 95% confidence intervals (CI) were calculated for each model. The areas under the receiver operating characteristic (ROC) curves (AUC) of the four models were calculated and compared via the DeLong test [16].

In addition, univariate and multivariate analyses were stratified by clinical risk groups and reported as Supplementary Material.

Finally, a nomogram for the prediction of ≥pT3a PCa findings at pathology was created. Multivariable logistic regression was used to build the nomogram, considering the categorical variables: risk group, ESUR EPE score, and PI-RADS v2 assessment category. Performance of the nomogram was assessed in terms of discrimination (Harrell’s c-index), which provides an estimate of the probability that the model will correctly identify patients who had ≥pT3a.

Statistical analysis was performed using the SAS software (SAS version 9.2) and R (R version 3.2.3) and its Hmisc and rms libraries (http://cran.r-project.org/).

## Results

**Fig. 1:**
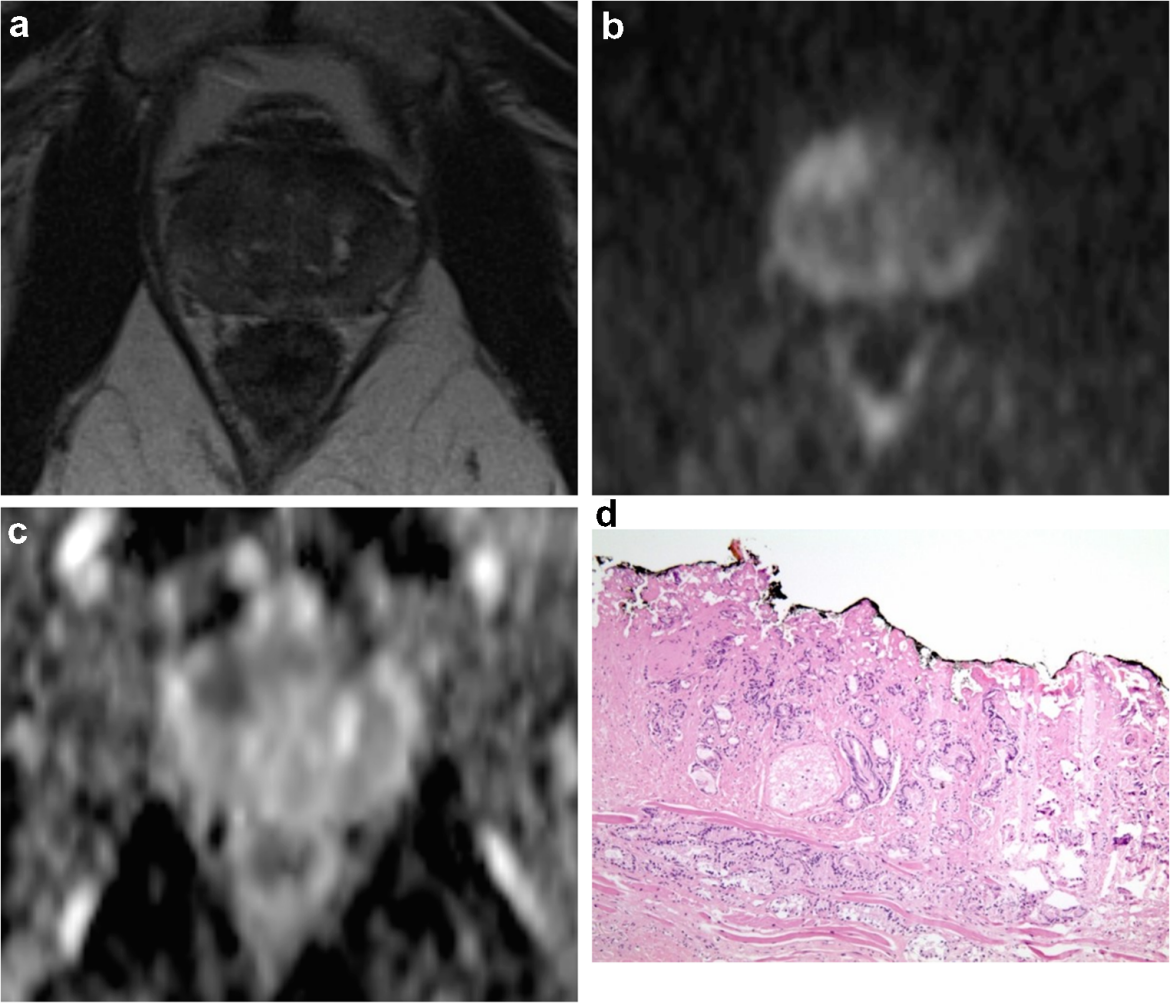
mp-MRI examination revealed an anterior, right lesion having a PI-RADS v2 score of 5 in a 66-year-old, clinically low-risk patient (cT1c, PSA 6.3, biopsy Gleason score 3 + 3) seen in (**a**) axial T2-weighted, (**b**) b1000 DWI, and (**c**) ADC map, where a pT3a, pathologic Gleason score 3 + 4 cancer was identified in (**d**) the histopathology specimen

**Fig. 2:**
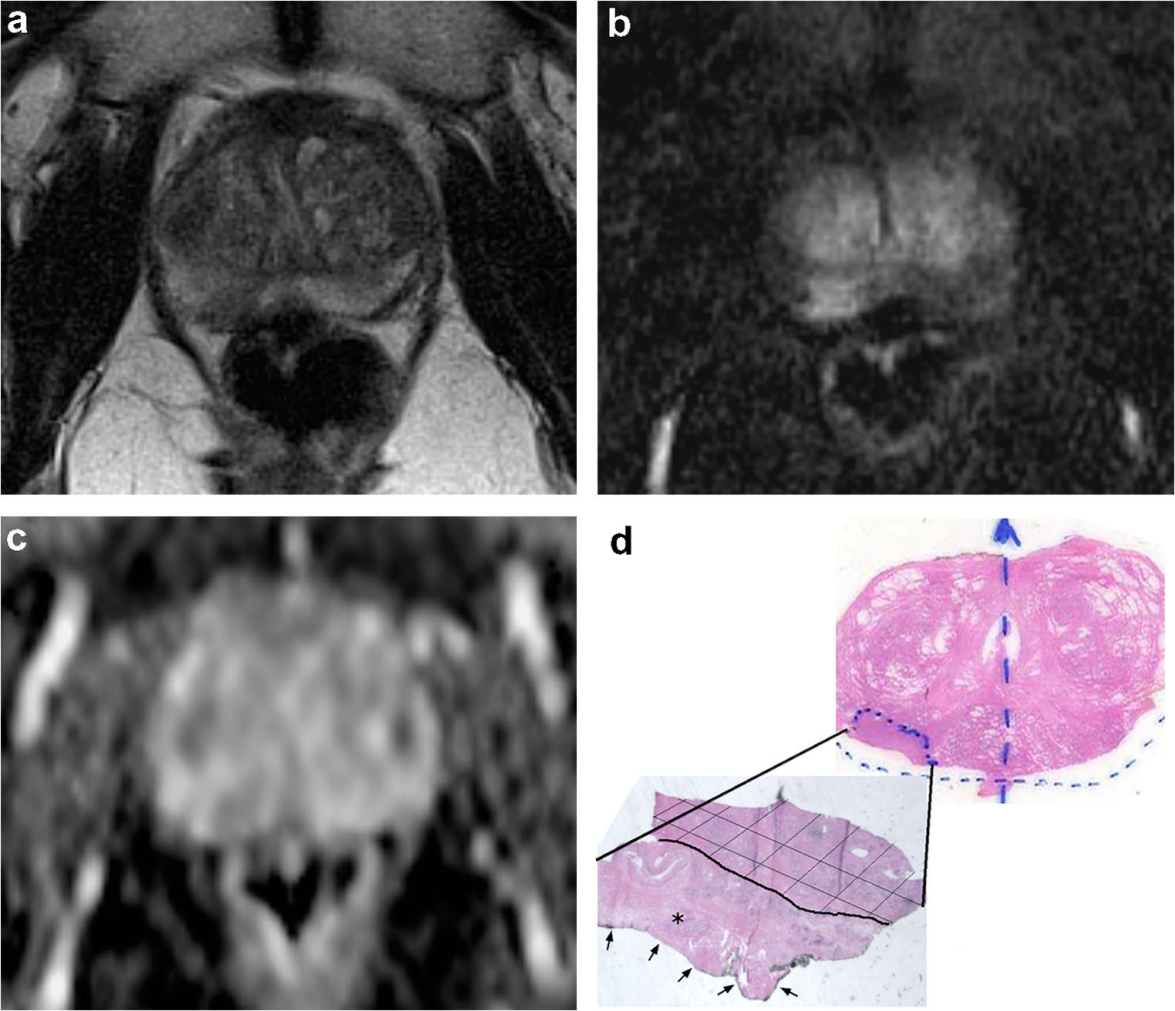
A right-sided, posterolateral lesion in a 65-year-old patient with a PI-RADS v2 score of 4 and EPE score of 2 at mp-MRI examination seen in axial (**a**) T2-weighted image, (**b**) subtracted DCE image, and (**c**) ADC map. At histology, the axial whole section of the apical portion of the prostate (**d**—upper right) without the posterolateral surgical margins sampled for intraoperative examination revealed a Gleason score 3 + 4 PCa. (**d**—lower left). In the intraoperative frozen section however, the PCa (hashed zone) was found to be pT3a, extending focally to the surgical margin (unhashed zone), including an extraprostatic site (*)

The demographic and clinical characteristics of the 301 patients in our cohort are described in Table 1. Based on pre-imaging clinical characteristics, there were 137 (45.5%) patients in the low-risk group (Fig. 1) and 164 (54.5%) in the intermediate/high-risk group (Fig. 2).

**Table 1:** Patient and tumor characteristics of the study population

	Low-risk group (*N* = 137)	Intermediate/high-risk group (*N* = 164)	Total (*N* = 301)
Age (years)	62.98 (± 6.98)	63.29 (± 6.96)	63.15 (± 6.96)
PSA (ng/ml)	5.98 (± 1.83)	10.89 (± 10.10)	8.66 (± 7.94)
Clinical stage			
cT1b	0 (0.00%)	2 (1.22%)	2 (0.66%)
cT1c	109 (79.56%)	82 (50.00%)	191 (63.46%)
cT2a	28 (20.44%)	59 (35.98%)	87 (28.90%)
cT2b	0 (0.00%)	1 (0.61%)	1 (0.33%)
cT2c	0 (0.00%)	9 (5.49%)	9 (2.99%)
cT3a	0 (0.00%)	11 (6.71%)	11 (3.65%)
Biopsy Gleason score			
3 + 3	137 (100.00%)	31 (18.90%)	168 (55.81%)
3 + 4	0 (0.00%)	70 (42.68%)	70 (23.26%)
4 + 3	0 (0.00%)	32 (19.51%)	32 (10.63%)
3 + 5	0 (0.00%)	3 (1.83%)	3 (1.00%)
4 + 4	0 (0.00%)	19 (11.59%)	19 (6.31%)
5 + 3	0 (0.00%)	2 (1.22%)	2 (0.66%)
4 + 5	0 (0.00%)	5 (3.05%)	5 (1.66%)
5 + 4	0 (0.00%)	2 (1.22%)	2 (0.66%)
ESUR EPE score			
1	27 (19.71%)	9 (5.49%)	36 (11.96%)
2	50 (36.50%)	27 (16.46%)	77 (25.58%)
3	30 (21.90%)	34 (20.73%)	64 (21.26%)
4	22 (16.06%)	48 (29.27%)	70 (23.26%)
5	8 (5.84%)	46 (28.05%)	54 (17.94%)
PI-RADS v2 score			
1	0 (0.00%)	0 (0.00%)	0 (0.00%)
2	2 (1.46%)	3 (1.83%)	5 (1.66%)
3	31 (22.63%)	6 (3.66%)	37 (12.29%)
4	39 (28.47%)	32 (19.51%)	71 (23.59%)
5	65 (47.45%)	123 (75.00%)	188 (62.46%)
Pathological stage			
pT2a	13 (9.49%)	5 (3.05%)	18 (5.98%)
pT2b	3 (2.19%)	7 (4.27%)	10 (3.32%)
pT2c	93 (67.88%)	61 (37.20%)	154 (51.16%)
pT3a	25 (18.25%)	68 (41.46%)	93 (30.90%)
pT3b	3 (2.19%)	23 (14.02%)	26 (8.63%)

Expressed as *N* (%) or mean (± standard deviation)

Low-risk group: PSA < 10 and Gleason score ≤ 3 + 3 and clinical stage ≤ 2a according to [3]; intermediate/high-risk group: the remaining patients

### Pathology findings

At final histology, pathology stage pT2c was the most frequent (154 patients; 51.2%), followed by pathology stages ≥pT3a, pT2a, and pT2b (Table 1).

### mp-MRI findings

The majority of the patients were in PI-RADS v2 assessment category 5 (62.5%), followed by categories 4 and 3; less than 2% of the patients were in category 2, and none in category 1 (Table 1). The distribution of PI-RADS assessment categories was truncated below (no PI-RADS v2 assessment category 1 findings) in both groups and relatively flat for the low-risk group. In contrast, it was skewed towards higher values in the intermediate/high-risk group (Table 1).

The distribution of ESUR EPE scores was shifted towards lower values in the low-risk group and towards higher values in the intermediate/high-risk group (Table 1).

### Univariate and multivariate associations

All evaluated clinical and tumor characteristics, except age, were significantly associated with pathological stage ≥pT3a at univariate analysis (Table 2). The same results were obtained on stratifying by clinical risk groups (Supplementary Table 2).

**Table 2:** Association of patient and tumor characteristics with ≥pT3a PCa: univariate analysis

	<pT3a	≥pT3a	*p* value*
Overall cohort (*N* = 301, prevalence of ≥pT3a 39.5%)			
Age (years)	62.64 (± 6.92)	63.93 (± 6.97)	0.11
Risk group			*< 0.0001*
Low	109 (79.56%)	28 (20.44%)	
Intermediate/high	73 (44.51%)	91 (55.49%)	
ESUR EPE score			
1–2	104 (92.04%)	9 (7.96%)	*< 0.0001*
3	47 (73.44%)	17 (26.56%)	
4–5	31 (25%)	93 (75%)	
PI-RADS v2 score			*< 0.0001*
1–2	5 (100.00%)	0 (0.00%)	
3	36 (97.30%)	1 (2.70%)	
4–5	141 (54.44%)	118 (45.56%)	

Expressed as *N* (%) or mean (± std dev)

*Nonparametric two-sample Wilcoxon test for age and chi-square test for categorical variables

Significant *p* values are in italics. Low-risk group: PSA < 10 and Gleason score ≤ 6 and clinical stage ≤ 2a according to [3]; intermediate/high-risk group: the remaining patients

Encompassing all risk groups, the performance of PI-RADS v2 assessment category for ruling out ≥pT3a PCa was described by SE, NPV, and LR− of 99%, 98%, and 0.04, respectively, and for ruling in ≥pT3a PCa by SP, PPV, and LR+ of 23%, 46%, and 1.28, respectively (Table 3).

**Table 3:** Sensitivity (SE), specificity (SP), positive and negative predictive values (PPV and NPV), and positive and negative likelihood ratios (LR+ and LR−) for predicting ≥pT3a, according to risk group, PI-RADS v2, and EPE score

Variables	*N*	Prevalence of ≥pT3a (%)	Ruling out performance			Ruling in performance		
			SE*	NPV*	LR−	SP*	PPV*	LR+
Risk group			76 (68–84)	80 (72–86)	0.39	60 (52–67)	55 (48–63)	1.91
ESUR EPE score			78 (70–85)	85 (79–90)	0.26	83 (77–88)	75 (66–82)	4.59
Low-risk group	137	20.4	64 (44–81)	91 (83–95)	0.40	89 (82–94)	60 (41–77)	5.84
Intermediate/high-risk group	164	55.5	82 (73–90)	77 (66–86)	0.24	74 (62–84)	80 (70–87)	3.17
PI-RADS v2			99 (95–100)	98 (87–100)	0.04	23 (17–29)	46 (39–52)	1.28
Low-risk group	137	20.4	96 (82–100)	97 (84–100)	0.12	29 (21–39)	26 (18–35)	1.37
Intermediate/high-risk group	164	55.5	100 (96–100)	100 (66–100)	0.00	12 (6–22)	59 (50–67)	1.14

*Expressed as % (95% CI)

*CI*, confidence interval. Low-risk group: PSA < 10 and Gleason score ≤ 3 + 3 and clinical stage ≤ 2a according to [3]; intermediate/high-risk group: the remaining patients

Encompassing all risk groups, the ESUR EPE score performance for ruling out ≥pT3a PCa was described by SE, NPV, and LR− of 78%, 85%, and 0.26, respectively, and for ruling in ≥pT3a PCa by SP, PPV, and LR+ of 83%, 75%, and 4.59, respectively (Table 3).

Looking at the results stratified by risk groups (Table 3), the performance of PI-RADS v2 assessment category and the performance of ESUR EPE score were quite similar to the above mentioned, with small increase of SE and PPV for both PI-RADS v2 assessment category and ESUR EPE score in the intermediate/high-risk group than in the low-risk group. A small decrease in SP, LR−, and LR+ was also observed for both PI-RADS v2 assessment category and ESUR EPE score in the intermediate/high-risk group in respect to the low-risk group.

The low clinical risk group was associated with a significantly lower probability of ≥pT3a PCa than the intermediate/high-risk group in all multivariable models (Table 4). Adding either the ESUR EPE score or the PI-RADS v2 assessment category to the clinical risk model (yielding model 2 and model 3, respectively) significantly improved the prediction of ≥pT3a PCa (model 2 AUC = 0.73, model 3 AUC = 0.86 relative to clinical risk model AUC = 0.68, both *p* < 0.0001). The full model (model 4, including PI-RADS v2 assessment category and ESUR EPE scores as well as clinical risk group) produced an AUC significantly higher than all the other models (Supplementary Table 4), but the OR relative to model 3 (PI-RADS v2 assessment category and clinical risk group) was not significant. Similar results were obtained in analyses stratified by clinical risk groups as presented in Supplementary Table 3.

**Table 4:** Modeling of patient and tumor characteristics for association with ≥pT3a PCa: multivariate analysis

	Model 1* OR (95% CI)	Model 2* OR (95% CI)	Model 3* OR (95% CI)	Model 4* OR (95% CI)
Overall cohort (*N* = 301, prevalence of ≥pT3a 20.4%)				
Risk group				
Intermediate/high	Reference	Reference	Reference	Reference
Low	*0.21 (0.12–0.35)*	*0.41 (0.22–0.77)*	*0.26 (0.15–0.44)*	*0.43 (0.23–0.81)*
ESUR EPE score				
4–5	–	Reference	–	Reference
3	–	*0.14 (0.07–0.27)*	–	*0.14 (0.07–0.28)*
1–2	–	*0.04 (0.02–0.09)*	–	*0.05 (0.02–0.12)*
PI-RADS v2 score				
4–5	–	–	Reference	Reference
1–2–3	–	–	*0.04 (0.01–0.32)*	0.21 (0.03–1.67)
AUC	0.682	0.856	0.730	0.862

*Model 1 is based on risk group; model 2 is model 1 adding 3 classes ESUR EPE score; model 3 is model 1 adding 2 classes PI-RADS v2 score; model 4 is model 2 adding PI-RADS v2 score. *OR*, odds ratio; *CI*, confidence interval; *AUC*, area under the curve. Significant ORs and 95% CI are in italics. Low-risk group: PSA < 10 and Gleason score ≤ 3 + 3 and clinical stage ≤ 2a according to [3]; intermediate/high-risk group: the remaining patients

ESUR EPE score and PI-RADS v2 assessment category were seen to be significantly correlated (Spearman’s correlation coefficient *r* = 0.55; *p* value < 0.0001).

### Nomogram

**Fig. 3:**
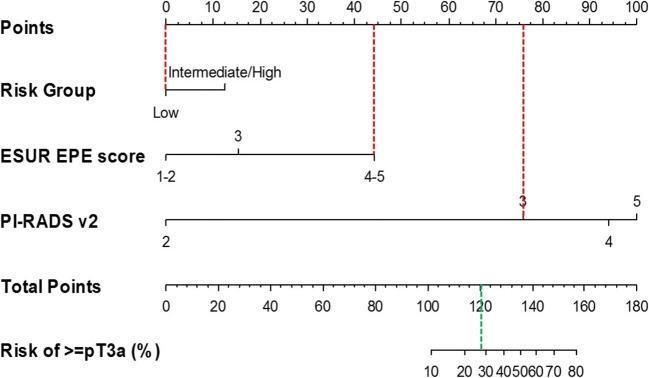
Nomogram for the prediction of the risk of ≥pT3a PCa based on clinical risk group, ESUR EPE score, and PI-RADS v2 score. For each patient variable (Risk Group, ESUR EPE score, and PI-RADS v2), a vertical line is drawn from the value on the bar for that variable to the upper scale of points (dotted red lines show that low-risk group corresponds to 0 points in the upper bar, ESUR EPE score 4–5 corresponds to about 45 points, and PI-RADS v2 = 3 corresponds to 75 points). The sum of these three points is then located on the scale indicating the “Total Points” (here: 0 + 45 + 75 = 120 total points), and a vertical line is drawn downwards (green dotted line). Where this line intersects, the scale for Risk of ≥pT3a (%) gives the percentage risk of ≥pT3a PCa. Values outside the indicated bar should be read as risk < 10% (for Total Points < 100) or risk > 80% (for Total Points > 160), respectively. In the example above, a subject in the low-risk group, with a ESUR EPE score of 4–5 and PI-RADS v2 score of 3 has about 28% risk of ≥pT3a PCa

The nomogram developed based on our cohort (Fig. 3) graphically displays the predicted risk of ≥pT3a PCa in relation to the combination of variables from the full model (model 4) examined in the multivariate analysis: clinical risk group, ESUR EPE score, and PI-RADS v2 assessment category. The C-index for our nomogram was 0.8538.

## Discussion

In our cohort of 301 operated prostate cancer patients, with a prevalence of 39.5% ≥pT3a PCa, we observed an overall very good performance of preoperative mp-MRI in ruling out ≥pT3a PCa using either PI-RADS v2 assessment category or ESUR EPE score (PI-RADS v2 SE 99%, NPV 98%, LR− 0.04; ESUR EPE score SE 78%, NPV 85%, LR− 0.26). Our results are comparable with those of Matsuoka et al [17] who recently evaluated PI-RADS v2 in assessing extracapsular extension and demonstrated a SE of 92.9% and a high NPV (96%), regardless of the risk group, in a cohort having a lower prevalence of ≥pT3a PCa (26.7%), but are in contrast with those of Gaunay et al [18] who saw comparatively low SE (8.3%) and NPV (81.5%), but high SP (97.8%) and PPV (93.3%) for the prediction of ≥pT3a PCa in a group of 74 operated prostate cancer patients where prevalence of ≥pT3a PCa was 32.4%.

As regards ESUR EPE evaluation, our results are comparable with those of Boesen et al [2], whose SE was 74% and LR− was 0.295 in a population of 87 operated prostate cancer patients with a ≥pT3a PCa prevalence of 37%. They are also similar with those of Somford et al [19], who had a SE of 58.2% and LR− of 0.47 in a cohort of 183 prostate cancer patients with a ≥pT3a PCa prevalence of 49.7%. Overall, our results indicate somewhat higher sensitivity and lower LR− than a recent meta-analysis of the mp-MRI in predicting ≥pT3a PCa that included 75 studies and 9796 patients, where the overall SE was 57%, SP 91%, and LR− 0.47 for detection of ≥pT3a PCa [5].

Both ESUR EPE score ≤ 2 and PI-RADS v2 assessment category ≤ 3 were associated with a 96% lower risk of ≥pT3a PCa than in patients with ESUR EPE score of ≥ 4 or PI-RADS v2 assessment category ≥ 4. The inclusion of ESUR EPE score to the clinical risk alone (model 2) or PI-RADS v2 assessment category to the clinical risk alone (model 3) significantly increased the AUC (from 0.68 to 0.86 (*p* < 0.0001) and to 0.73 (*p* < 0.0001), respectively). Similar results were also obtained in both the multivariate analyses stratified by clinical risk groups (see Supplementary Material). The combined addition of both ESUR EPE score and PI-RADS v2 assessment category to clinical risk (model 4) yielded a slight but statistically significant further increase in AUC if compared with model 2 (from 0.856 to 0.862 (*p* = 0.04)).

The similarity of performance in excluding ≥pT3a PCa when adding either or both ESUR EPE score and PI-RADS v2 assessment category to the clinical risk suggests a close relationship between these two forms of assessment.

We found a correlation (*r* = 0.55) between PI-RADS v2 assessment categories and ESUR EPE score as indicators of the presence of ≥pT3a PCa. It could be expected due to the criteria that define the categories in the respective scales.

Explicit visualization of extraprostatic extent on mp-MRI is a criterion for assigning a value of 5 under both PI-RADs v2 assessment category and ESUR EPE score criteria, resulting in a concentration of ≥pT3a lesions in these categories by construction. Even in the absence of visible extraprostatic extent, it has been demonstrated that the risk of ≥pT3a PCa is associated with tumor contact length, with a risk rising from below 10% when contact is < 10 mm, to about 40% when contact is 15 mm, and over 60% when contact is > 20 mm [20]. Moreover, it is recognized that low ADC values in index lesions, a second condition leading to increased PI-RADS v2 assessment category values, are correlated with a higher Gleason score that is in turn directly correlated with tumor aggressiveness and consequently with risk of ≥pT3a PCa [21].

We note that our multivariate analyses suggest that the ESUR EPE score is a slightly stronger factor in predicting ≥pT3a PCa than PI-RADS v2 assessment category. Unfortunately, the evaluation of ESUR EPE score is not as well standardized as PI-RADS v2 scoring system and remains subjective, with low inter-observer agreement for the assessment of pT3a having been reported by some authors [22, 23]. Thus, some effort towards standardization of ESUR EPE score evaluation would appear to be a valuable contribution to clinical practice. Until ESUR EPE score evaluation can be reproducibly performed, the use of PI-RADS v2 assessment categories provides a smaller, but significant, improvement in the exclusion of ≥pT3a PCa that can be widely adopted.

The association between PI-RADS v2 and ≥pT3a PCa has immediate clinical implications for patient care and management of patients. As regards patient counseling, in particular for functional preservation treatments, PI-RADS v2 assessment categories 1, 2, and 3 effectively exclude ≥pT3a PCa (risk notably less than 10%) and could promote suitability in program of active surveillance. Conversely, the presence of PI-RADS v2 assessment categories > 3 can inform the decision to avoid a nerve-sparing approach in the site of tumor contact with capsule during radical prostatectomy, or to perform in that site intraoperative frozen sections [11].

Numerous nomograms have been developed for the prediction of ≥pT3a, including the Partin tables and Memorial Sloan Kettering Cancer Center (MSKCC) nomograms [24, 25], but these nomograms do not include the diagnostic contribution of mp-MRI. Feng et al [26] compared the predictive accuracy of MRI and clinical models (Partin tables and MSKCC nomogram) for pT3a finding a small improvement in diagnostic accuracy after addition of MRI to the models (AUC for Partin tables and MSKCC of 0.85 and 0.86, respectively, increased to 0.93 and 0.94 after addition of MRI). Recently, Weaver et al [27] examined the incremental value of prostate MRI when used in combination with the currently available preoperative risk stratification tool, the MSKCC nomogram. They suggest that the use of prostate MRI as a predictive tool should be performed in combination with the clinical risk stratification models.

The nomogram created from our cohort (Fig. 3) had a good calibration index (0.8538), but it needs clinical validation and development in other larger cohorts. Notably, while PI-RADS v2 assessment category resulted in a not significant OR in the full multivariate model, it has a strong influence on the scores that are obtained using the nomogram. In particular, whereas the ESUR EPE score can contribute a maximum of 45 points, the PI-RADS v2 assessment category can contribute up to 100 points. The maximum value obtained from ESUR EPE score and group of risk is about 55 points that means a risk of ≥pT3a PCa lower than 10%, but PI-RADS v2 assessment category plays a decisive role in increasing the estimated risk for higher ≥pT3a PCa, which can rise to over 80% depending on PI-RADS v2 assessment category.

A key limitation of our study relates to the fact that all patients had undergone prostatectomy, of whom 54% were of intermediate/high risk with the overall prevalence of ≥pT3a PCa being 39.5%. In addition, treatment decisions were influenced by the original reporting of the mp-MRI under PI-RADS v1, and thus the cohort may be subject to possible over- or underdiagnosis associated with that reporting system. Due to patient choice to undergo surgery after positive biopsy, there is a relatively high representation of low-risk patients in our cohort (46%), but the proportion of PI-RADS v2 scores ≤ 3 patients is low (15%). Thus, our results are most applicable to a population with known prostate cancer, particularly intermediate- to high-risk disease, and cannot necessarily be extrapolated to screening in the general population, where the expected prevalence of PCa ranges from 5% at age < 30 years to 59% by age > 79 years [28].

As well, we used consensus reporting by two radiologists to improve accuracy of PI-RADS v2 category determinations. This may limit the applicability of our results for single readers in clinical practice. Reports indicate that inter-reader reproducibility of PI-RADS v2 tends to be moderate and experience dependent [29, 30]. We would therefore expect that expert single readers would have similar performance to that reported in the present study.

Another limitation is that in our investigation of the prediction of ≥pT3a PCa on a per-patient basis, there was no direct comparison between the regions suspected of cancer based on mp-MRI and the tumor focus detected at whole-mount histopathologic examination. The data available retrospectively did not allow analysis at the lobe level to be performed in this study; a future work evaluating PI-RADS category scores and adjacent EPE at a per lesion level would provide further insight into the local depiction of ≥pT3a PCa and should correlate the location of the suspicious lesions to the pathologic stage.

Lastly, the nomogram developed based on our cohort must be validated and tested for predictive ability in a larger population of patients, and in other clinical centers.

In conclusion, the addition of PI-RADS v2 assessment category to clinical risk parameters improves the prediction of ≥pT3a PCa, and thus risk stratification. In particular, PI-RADS v2 assessment categories of 1 to 3 are useful for excluding ≥pT3a PCa with a NPV of 98%. This is important for clinical practice and for appropriate patient counseling; as such patients can be considered as candidates for less invasive approaches (active surveillance, nerve-sparing surgery, or prostate-only radiotherapy). The ESUR EPE score should be better standardized to make full use of the available information in evaluating prostate disease. A nomogram that combines clinical and mp-MRI parameters for prediction of ≥pT3a PCa has been developed based on our cohort and requires validation in larger and different populations prior to use in clinical practice.

## Electronic supplementary material

ESM 1 (DOCX 34.6 kb)

## Abbreviations

EAU: European Association of Urology
EPE (≥pT3a): Extraprostatic extension
ESUR: European Society of Urogenital Radiology
LR−: Negative likelihood ratio
LR+: Positive likelihood ratio
PCa: Prostate cancer
RARP: Robotic-assisted radical prostatectomy
SE: Sensitivity
SP: Specificity

## Footnotes

1 Herein, we use the pathology stage ≥pT3a PCa to avoid possible confusion with the European Society of UroRadiology extracapsular extension (ESUR EPE) score [3].
