# Supplementary material for: Low PI-RADS assessment category excludes extraprostatic extension (≥pT3a) of prostate cancer: a histology-validated study including 301 operated patients
Source: Eur Radiol. 2019 Mar 18;29(10):5478–87. doi: 10.1007/s00330-019-06092-0 (PMC6719329; doi:10.1007/s00330-019-06092-0)
Supplement: Supplementary file 1 — (DOCX 34.6 kb) [file 330_2019_6092_MOESM1_ESM.docx]

**Supplementary Table 1. Multiparametric MRI Acquisition Parameters**

|  | **T2WI** | **DWI** | **DCE*** |
| --- | --- | --- | --- |
| Acquisition plane | Axial; Coronal; Sagittal | Axial | Axial |
| Pulse sequence type | Turbo spin-echo | Echo-planar imaging | 3D gradient echo |
| Turbo factor | 23 | 60 | - |
| b-values (s/mm^2^) | - | 0, 500, 1000 | - |
| Section thickness | 3 | 4 | 4 |
| Intersection gap | 0.3 | 0.4 | 0 |
| Number of sections | 18 | 20 | 20** |
| Acquisition duration (min:sec) | 5:17 | 3:03 | 4:10 |
| Phase-encoding direction | RL; RL; AP | AP | AP |
| Field of view (mm) | 190x190 | 290x200 | 280x254 |
| Acquisition matrix | 320x320 | 128x66 | 320x228 |
| Number of repetitions | 5 | 8 | 1; 25 dynamics |
| TE/TR (ms) | 114/4440 | 69/3100 | 4.7/7.3 |
| Flip angle (°) | 90 | 90 | 25 |
| Bandwidth (Hz/pixel) | 140 | 1562 | 430 |

* T1-weighted, contrast agent (gadopentetate dimeglumine, 0.1 mmol / kg) was injected after the third dynamic acquisition at a flow rate of 3mL/s

** interpolated from 12

Note: AP = anterior-posterior, DCE = dynamic contrast-enhanced, DWI = diffusion weighted imaging, RL = right-left, TE/TR = echo time/repetition time, T2WI = T2-weighted imaging

Statistical Analysis by Clinical Risk Subgroups:

Reflecting routine clinical practice, we further evaluated the association between clinical and radiological variables with pathological stage ≥pT3a at univariate analysis, stratifying by clinical risk groups. Similarly, the unconditional logistic regression models based on ESUR EPE score and PI-RADS v2 assessment category were also evaluated for the clinical risk sub-groups.

Univariate analysis stratified by clinical risk groups confirmed results from overall population. ESUR EPE score and PI-RADS v2 assessment category resulted statistically associated with ≥pT3a PCa (Supplementary Table 2).

The results of multivariate analysis in the clinical subgroups were similar to those in the cohort as a whole. For the low-risk groups, the inclusion of both of ESUR EPE score and PI-RADS v2 assessment categories in the multivariate model, led to the greatest performance in predicting ≥pT3a PCa (AUC =0.81) if compared with model with only ESUR EPE score (AUC=0.787) or only PI-RADS v2 assessment category (AUC=0.629) (Supplementary Table 3).

For the intermediate/high-risk group there were no subjects with PI-RADS v2 assessment category ≤3 and ≥pT3a PCa, so that ORs for PI-RADS v2 assessment category were not estimable in both model with only PI-RADS v2 assessment category and full model (Supplementary Table 3).

**Supplementary Table 2. Association of patient and tumor characteristics with ≥pT3a PCa: univariate analysis stratified by risk groups**

|  | **<pT3a** | **≥pT3a** | ***p*-value*** |
| --- | --- | --- | --- |
| **Low-risk Group** *(N=137, prevalence of ≥pT3a 20.4%)* | | | |
| **Age** (yrs) | 62.84 (±6.54) | 63.54 (±8.58) | 0.40 |
| **ESUR EPE Score** |  |  |  |
| 1-2 | 71 (92.21%) | 6 (7.79%) | **<0.0001** |
| 3 | 26 (86.67%) | 4 (13.33%) |  |
| 4-5 | 12 (40.00%) | 18 (60.00%) |  |
| **PI-RADS v2 Score** |  |  | **0.002** |
| 1-2 | 2 (100.00%) | 0 (0.00%) |  |
| 3 | 30 (96.77%) | 1 (3.23%) |  |
| 4-5 | 77 (74.04%) | 27 (25.96%) |  |
| **Intermediate/High-risk Group** *(N=164, prevalence of ≥pT3a 55.5%)* | | | |
| **Age** (yrs) | 62.34 (±7.49) | 64.05 (±6.45) | 0.22 |
| **ESUR EPE Score** |  |  |  |
| 1-2 | 33 (91.67%) | 3 (8.33%) | **<0.0001** |
| 3 | 21 (61.76%) | 13 (38.24%) |  |
| 4-5 | 19 (20.21%) | 75 (79.79%) |  |
| **PI-RADS v2 Score** |  |  | **0.001** |
| 1-2 | 3 (100.00%) | 0 (0.00%) |  |
| 3 | 6 (100.00%) | 0 (0.00%) |  |
| 4-5 | 64 (41.29%) | 91 (58.71%) |  |

Expressed as N (%) or Mean (±std dev).

*non-parametric two-sample Wilcoxon test for age and chi square test or Fisher exact test for categorical variables, as appropriate;

Note: significant p-values are in bold. Low-risk Group: PSA<10 and Gleason Score≤3+3 and Clinical Stage≤2a according to (3); Intermediate/High-risk Group: the remaining patients.

**Supplementary Table 3. Modeling of patient and tumor characteristics for association with ≥pT3a PCa: multivariate analysis stratified by risk groups**

|  | **Model 2’***  **OR (95%CI)** | **Model 3’***  **OR (95%CI)** | **Model 4’***  **OR (95%CI)** |
| --- | --- | --- | --- |
| **In Low-risk Group** *(N=137, prevalence of ≥pT3a 20.4%)* | | | |
| **ESUR EPE Score** |  |  |  |
| 4-5 | Reference | - | Reference |
| 3 | **0.10 (0.03-0.37)** | **-** | **0.11 (0.03-0.41)** |
| 1-2 | **0.06 (0.02-0.17)** | **-** | **0.08 (0.03-0.26)** |
| **PI-RADS v2 Score** |  |  |  |
| 4-5 | - | Reference | Reference |
| 1-2-3 | - | **0.09 (0.01-0.68)** | 0.25 (0.03-2.12) |
| ***AUC*** | *0.787* | *0.629* | *0.811* |
| **In Intermediate/High-risk Group** *(N=164, prevalence of ≥pT3a 55.5%)* | | | |
| **ESUR EPE Score** |  |  |  |
| 1-2 | Reference | - | Reference |
| 3 | **0.16 (0.07-0.37)** | **-** | **0.16 (0.07-0.37)** |
| 4-5 | **0.02 (0.01-0.08)** | **-** | **0.03 (0.01-0.12)** |
| **PI-RADS v2 Score** |  |  |  |
| 4-5 | - | Reference | Reference |
| 1-2-3 | - | N.E. | N.E. |
| ***AUC*** | *0.809* | *N.E.* | *0.812* |

* Model 2’ based on 3 classes ESUR EPE score; model 3’ is based on 2 classes PI-RADS v2 score; model 4’ is based on both ESUR EPE score and PI-RADS v2; corresponding to models 2 – 4 in Table 4a without clinical risk group.

Note: Significant ORs and p-values are in bold. Low-risk Group: PSA<10 and Gleason Sore≤3+3 and Clinical Stage≤2a according to (3); Intermediate/High-risk Group: the remaining patients. N.E. not evaluable insufficient membership in subgroups. OR: Odds Ratio. CI: Confidence interval. AUC: Area under the curve.

**Supplementary Table 4. P-values for the difference between AUC of Models 1 to 4***

|  | **Model 1** | **Model 2** | **Model 3** | **Model 4** |
| --- | --- | --- | --- | --- |
| **Model 1** |  | **<0.0001** | **<0.0001** | **<0.0001** |
| **Model 2** |  |  | **<0.0001** | **0.04** |
| **Model 3** |  |  |  | **<0.0001** |
| **Model 4** |  |  |  |  |

* Model 1 is based on Group Risk; model 2 is model 1 adding three classes (1,2 vs. 3 vs .4,5) of ESUR EPE score; model 3 is model 1 adding two classes (≤3 vs. ≥4) of PI-RADS v2 assessment category; model 4 is model 2 adding two classes (≤3 vs. ≥4) of PI-RADS v2 assessment categories.

Note: Significant p-values are in bold. AUC: Area under the curve.
